# To dystrophin and beyond: an interview with Louis Kunkel

**DOI:** 10.1242/dmm.043018

**Published:** 2019-12-12

**Authors:** Louis M. Kunkel

**Keywords:** Genetic disease, Interview, Neuromuscular disease

## Abstract

Louis Kunkel has devoted his career to understanding the causes, mechanisms and treatment of muscular dystrophies. Dr Kunkel is the past Director of the Genomics Program at Boston Children's Hospital and Professor of Genetics and Pediatrics at Harvard Medical School. In this interview, he talks about his discovery of dystrophin, including patients in preclinical research, and bearded irises.


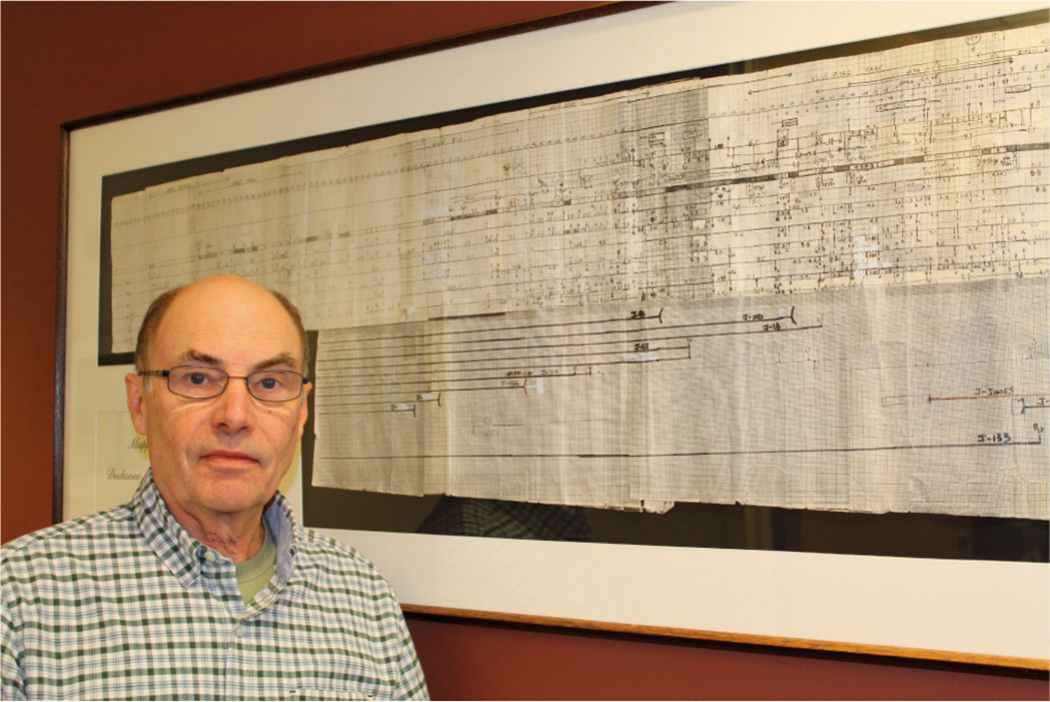


**Introduction**

Louis M. Kunkel received his PhD from Johns Hopkins University, where he trained in Victor McKusick's human genetics program under the mentorship of Samuel Boyer and Kirby Smith. There, he described sequences specific to the human Y chromosome. He continued his work on sex chromosomes as a postdoc at the University of California, San Francisco. In the 1980s, Lou and his team identified dystrophin, a key muscular protein that, when mutated, results in Duchenne and Becker muscular dystrophies. In the three decades that followed, Lou built upon his seminal discovery, providing key knowledge on the genetics and pathological mechanisms of many neuromuscular disorders. He is a thoughtful and generous mentor, and he proudly includes patients in his group's research, aiming at fulfilling the promise of personalised medicine for genetic diseases.

**Both your father [immunologist Henry G. Kunkel] and grandfather [plant pathologist Louis O. Kunkel] were academics. Were they your main inspiration to pursue a scientific career?**

I was a bit inspired by both my grandfather and father; I grew up in a house where science was regularly the topic of conversation. We regularly talked about science and nature. But my biggest influence was summer jobs in labs as an undergraduate student. One of my biggest influences was a summer placement in Alexander Bearn's lab in New York and I got to visit the Moore clinic at Johns Hopkins in Baltimore, where Victor McKusick held court. Victor asked me a question and, even though I was an undergraduate, I was able to answer. This ultimately motivated me to attend graduate school at Johns Hopkins and join Victor's human genetics program. So I suppose my biggest influence was meeting Victor and him asking me to join his graduate program.

**You are best known for your discovery of dystrophin and your career-long dedication to muscular dystrophy research. Can you tell us how it all started?**

Serendipitously. I trained in human genetics with Samuel Boyer and Kirby Smith, working on the Y chromosome. I was able to isolate DNA sequences that were specific to the human Y chromosome, using a subtractive hybridization approach. Due to this work, I was advised to pursue postdoctoral training in a different organism. So I left Hopkins and became a postdoctoral fellow at the University of California in San Francisco, where I worked on the *Drosophila* Y chromosome, but I needed to leave California after only 2 years. I returned to Boston and joined the late Samuel Latt's group for another postdoctoral fellowship. He took me in for a year, after which I was required to secure my own funding.

Since we were working on the human X chromosome, I decided to apply for a fellowship from the Muscular Dystrophy Association (MDA). My proposal was to map and characterize the Duchenne gene, and this fellowship was actually my starting point in muscular dystrophy research. So, sort of a serendipitous start.

**In a way, this sounds like what many young scientists experience nowadays, where funding availability often steers one's research?**

It is quite common now. To survive in academia, you need support beyond public or government funding, and charitable organizations have an important role. I believe it's much harder to get philanthropic funding now than it was back when I first applied for my fellowship. There's greater competition. We train so many brilliant young scientists who inevitably end up competing with each other for limited resources. And public investment in research through the NIH [National Institutes of Health] has not kept pace with the increased numbers of scientists who compete for funding.

“We train so many brilliant young scientists who inevitably end up competing with each other for limited resources.”

Science drives progress, even economic progress, and substantial investment is key to this. China is an excellent example of a country where the government placed research as one of its highest priorities.

**Switching back: many neuromuscular diseases have an important cognitive and neurodevelopmental component. Can you discuss your work on this topic?**

We are indeed working on cognitive impairment in Duchenne patients and are developing a mouse model system where we're knocking out dystrophin specifically in the brain. We plan on studying the behaviour of these knockout mice and try to find parallels with the cognitive and behavioural symptoms of human patients. The nervous system's involvement in Duchenne is a relatively new area of intense research, because the cognitive aspect gets masked by the enormous effect that lack of dystrophin has on the patient's muscles. However, as therapies such as exon skipping and gene therapy are starting to treat the muscular disorder, we are beginning to see more of the neurological and cognitive consequences of these mutations.

**You work on a number of model systems, which are all very different from each other. As one research group, how do you manage the specifics of each experimental platform?**

A lot of my postdocs, students and technicians have experience and the proper skills to work with different animal models – cells, mice and zebrafish. Actually, many prefer working with fish, as they are easier to handle and experimental turnaround is faster, and the cost of zebrafish maintenance is much smaller than for mice. However, we do use mice to validate findings from fish experiments.

**Looking back at your research, can you think of a moment or a result that dramatically shifted your perspective – a Eureka moment of sorts?**

Most science builds slowly, one finding leading to the other, so Eureka moments are incredibly rare. For me, the closest I got was when Tony Monaco, a graduate student in my lab, called me at home to tell me he was pretty sure he had the Duchenne gene. My wife had just given birth to our oldest daughter and we had just brought her home when I got the call. So that was quite a memorable moment in time. I couldn't wait to get into the lab to see the results.

**Can you tell us a bit more about the hands-on process of hunting for a disease gene?**

We based our work on previous knowledge from genetic linkage analyses, so we roughly knew where on chromosome X the candidate gene is likely to be located. We had to do chromosome walking, which was a laborious but necessary method pre-Human Genome Project, assembling contigs and comparing the wild-type DNA to that of patients who had deletions. We sequenced every cDNA clone by hand and manually typed every sequence into a computer. So it was very different from how things are done now.

**What you are describing sounds like a completely different world**

It sort of was. When I was a postdoc in California, we had to make our own restriction enzymes. That was the role of a postdoc in the lab – everybody had to make at least one restriction enzyme. Now you can order them and they arrive on your bench the next day. And to further drive this point home, I was a graduate student when Fred Sanger published his sequencing method. I can still remember trying to explain the chain termination technology to my mentor at the time.

Even now, with all the genomics technology and off-the-shelf reagents available, we need to keep innovating to come up with new ways to find solutions, and you still need to have good hands and great attention to detail. If you don't set up your PCR or cloning reaction right, no matter if the mix is off-the-shelf or ‘homemade’, the reaction won't work. So science is not necessarily easier today than it was four decades ago.

**Remarkably, you also actively include patients in your research and are actively involved in patient outreach, even though you are not a clinician. What first motivated you for this?**

It's hard to work on diseases like Duchenne muscular dystrophy and facioscapulohumeral muscular dystrophy (FSHD) and not get involved with patients. Those are rare diseases, and accruing clinical samples for research is not trivial. When my group first identified dystrophin, we were the ones who ran diagnostic tests for Duchenne. I've been part of patient advocacy groups, like the MDA and the FSHD Society, for many years. I think it also helps researchers to stay motivated; for example, when my lab members get to meet some of the patients and see the impact their work has on real people. Makes everyone work a little bit harder.

“It's hard to work on diseases like Duchenne muscular dystrophy and FSHD and not get involved with patients.”

**Do you have any advice for your fellow scientists who want to get involved with patients?**

I think in the neuromuscular disease area, many scientists already actively involve patients in their research. Many of my lab alumni remain actively involved with patient advocacy. The diseases we study are rare and there are few patients. So participating in advocacy groups with active involvement from researchers perhaps gives them a sense of comradery and a louder voice.

**We recently interviewed Elizabeth McNally, who is a former postdoc of yours and a fellow leading figure in the field, and we asked her about her leadership and whether she takes intentional steps to support minorities and women in science. So it's only fair that we ask you the same**

I try my best to support colleagues from minority groups and women. I think I have been much more successful in helping foster women than minority scientists, but it's definitely something I'm mindful of. When I review applications for open positions in my lab, most of the applicants are women – I do not know why that is, but I think by now I trained more women than men and am proud of their success. Maybe I have a ‘good karma’ for mentoring women [laughs], and I have three daughters and no sons. I also think being a father to daughters has changed my perspective and has made me more sensitive to the issues women still face every day that men perhaps do not.

**Can you tell us something about you that people would be surprised to learn?**

I love gardening, and did ‘garden genetics’ with my father – we bred German bearded irises. Many of the crosses we made still bloom in my daughter's garden. Irises are propagated vegetatively, and I still have an iris cross that my dad got from his mother – my grandmother – more than 70 years ago, and they are still in the family.

**This is a very interesting hobby. Any lessons from breeding irises that you were able to transplant to your work in the lab?**

You learn patience. When you cross the irises, you need to wait for the seeds to develop in a pod, dry them and then wait until the following spring to plant the seeds. So it takes 2-3 years to see the new blossoms bloom. Experimental patience is very true in science, too. Things don't move very fast. Probably 90% of the work we do in the lab is just build-up until you get to see the final result – the figurative iris blooming.

“[…] it takes 2-3 years to see the new blossoms bloom. Experimental patience is very true in science, too.”

**Is experimental patience something you teach your trainees?**

Not explicitly, but I do stress the importance of patience when we discuss plans and results. However, recognizing opportunities and pursuing those is just as important in research.

**Aside from gardening, what do you enjoy doing outside of the lab?**

I love the outdoors and have been an avid runner for about 40 years now. I'm slowing down now, but am still passionate about it.

